# Changing to Impress: Testing a Mediation Model from Instagram Self-presentation to Cosmetic Surgery

**DOI:** 10.1007/s00266-023-03501-0

**Published:** 2023-07-29

**Authors:** Tommaso Galeotti, Claudia Marino, Natale Canale, Luca Scacchi, Alessio Vieno

**Affiliations:** 1 Dipartimento di Psicologia dello Sviluppo e della Socializzazione, Università degli Studi di Padova, Via Venezia, 8, 35131 Padova, Italy; 2 Dipartimento di Scienze Umane e Sociali, Università della Valle d’Aosta, Aosta, Italy

**Keywords:** Instagram use, Self-presentation, Objectification theory, Photo manipulation, Cosmetic surgery

## Abstract

**Background:**

This study examined the relationship between false self-presentation on Instagram and consideration of cosmetic surgery through the mediating role of body image control in photos (BICP), photo manipulation, and body shame. We predicted that false self-presentation on Instagram was indirectly associated with cosmetic surgery intentions through the aforementioned constructs.

**Methods:**

A total of 504 young Italian adults (28.2% males, 18–30 years) completed an online survey. They completed a questionnaire containing the Self-presentation on Instagram Questionnaire, the Body Image Control in Photos Questionnaire—revised, the Photo Manipulation Scale, the Objectified Body Consciousness Scale, and the Acceptance of Cosmetic Surgery Scale. The pattern of associations between the constructs was analyzed via path analysis.

**Results:**

The results show that false self-presentation on Instagram was associated with photo manipulation, both directly and indirectly, through BICP. Furthermore, photo manipulation was linked to body shame, but neither of them was associated with cosmetic surgery intentions. Finally, false self-presentation on Instagram was associated with the consideration of cosmetic surgery only through the mediation of BICP.

**Conclusion:**

Findings indicate that self-presentation styles might affect Instagram photo behaviors and individuals’ cosmetic surgery intentions, suggesting that surgeons should fully examine patients’ motivations before providing them with services. Furthermore, intervention programs encouraging users to present a more authentic version of themselves online might reduce the risk of self-objectification and reduce the consideration of procedures aimed at modifying one's body for purely aesthetic reasons.

**Level of Evidence V:**

This journal requires that authors assign a level of evidence to each article. For a full description of these Evidence-Based Medicine ratings, please refer to the Table of Contents or the online Instructions to Authors www.springer.com/00266.

## Introduction

During the last decade, social media has shaped our culture by glamorizing certain lifestyles, looks, and behaviors. Instagram, with its 1.2 billion monthly active users worldwide [1], played a prominent role in this process. The platform is an environment for stylized self-presentation [2]: that is, a place in which algorithmic interactions reward users who show a happy, constructed version of their life [3]. This process encourages creators to modify their self-presentations to impress others [4], often putting forth a false, more polished version of themselves. To achieve this result, users need to check for flaws in their photos before sharing them, thus taking an observer’s point of view in a process similar to self-objectification [5, 6]. According to objectification theory [7], self-objectification is expressed as the constant monitoring of one’s appearance and might lead to body shame and dissatisfaction [8, 9]. In turn, body shame might encourage people to search for methods to alleviate their discomfort [10], such as cosmetic surgery [11]. In contrast to reconstructive surgery, cosmetic procedures are surgical and non-surgical medical treatments designed to “enhance” physical appearance [12]. Although these procedures are in large part safe [12], there is still a risk of complications, especially if they are combined or repeated [13]. This study aimed to better understand the relationship between false self-presentation on Instagram and cosmetic surgery intentions by testing a mediation model embedded in objectification theory.

### False Self-presentation on Instagram

Instagram is a social media platform that incentivizes users to control and modify their self-presentation through the use of photographs [14, 15]. By posting photos of themselves online, people can express their personas and infer whether they are socially accepted from the number of likes and comments they receive [16]. Because images conforming to beauty ideals are easily recognized and liked online, users might enhance their self-presentation to feel validated [17]. The awareness of being evaluated by an audience, whether real or imagined, can be associated with self-objectification [18], a construct that refers to the assumption of an externalized point of view of one’s own body [7] and that is generally operationalized with body surveillance [19]. Previous research has highlighted that people with high self-monitoring tend to present a false version of themselves online to impress others and receive better feedback [20]. Thus, users might try to show a false self-image on Instagram to impress others by becoming more worried about how their body image is depicted in photos [21, 22] and digitally manipulating them [23, 24].

In this regard, Pelosi et al. [25] developed a self-report measure called Body Image Control in Photos (BICP) Questionnaire that evaluates the self-presentation of body image through photographs on social media. BICP refers to people’s understanding of their photo quality, concerns about how they are portrayed, and the strategies used in taking, choosing, and sharing pictures [22, 25]. This heightened attention to body appearance is conceptually similar to self-objectification, which has been linked to BICP [21].

### Photo Manipulation and Body Shame

Photo manipulation is the perfect way to control one’s body image online. Its use has been defined as a modern form of body surveillance [6] because users focus on their bodies from an external point of view and check for potential flaws [26]. Noticing these imperfections, people might realize that they are not able to reach their idealized version, thus feeling ashamed of their aspect [9]. Furthermore, easy accessibility and usage of manipulation tools might normalize their adoption, making such use a requirement before posting [27]. Thus, app affordances create a different but realistic version of the users, helping them visualize potential new looks. The fact that these makeovers are easily accessible to their virtual person might make people think that transformations are obtainable without difficulty for their real persona as well, and encourage them to consider cosmetic enhancements [28]. In this regard, there is already anecdotal evidence that people ask plastic surgeons to look like their selfies [29, 30]. Previous research has demonstrated that photo manipulation is associated with higher body dissatisfaction [31–33] and cosmetic surgery intention [34–36].

To this regard, body shame represents the negative feeling experienced when a person falls short of the idealized cultural standards of beauty. Even though this emotional state originates from the person’s body, people are driven to feel shame about their value as a whole [37]. Thus, to alleviate their discomfort, individuals might feel the need to pursue these internalized standards by undertaking procedures focused on body modification, such as cosmetic surgery [38, 39]. Seeing part of one’s own body as defective plays into the medicalization of appearance, which, in turn, popularizes cosmetic surgery since it shows these ideals as easily attainable [40, 41]. Previous research has indicated that body shame positively predicts cosmetic surgery acceptance for both personal and social reasons [11, 42].

### Current Study

**Fig. 1:**
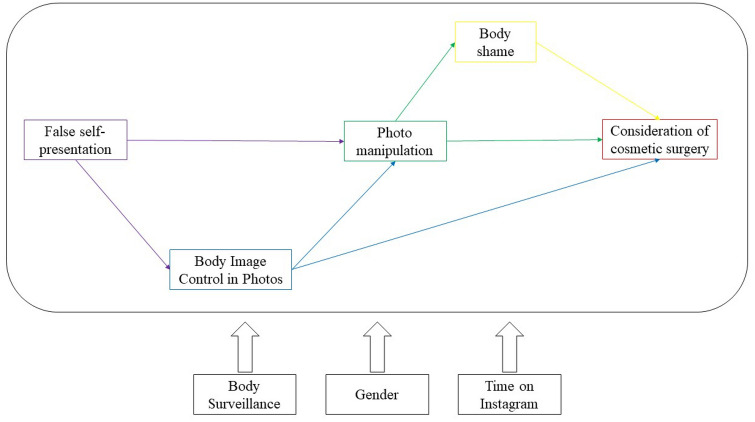
Hypothesized conceptual model

In the current study, we tested the relationship between false self-presentation on Instagram and consideration of cosmetic surgery through the mediating role of BICP, photo manipulation, and body shame. Our conceptual model is illustrated in Fig. 1.

Based on the literature reviewed above, our specific hypotheses were as follows:

1. False self-presentation on Instagram would be directly associated with photo manipulation and indirectly through BICP. That is, individuals who try harder to impress others would manipulate their photos. Moreover, they would engage in higher levels of body image control behaviors before sharing their photos, which, in turn, would result in higher photo manipulation behaviors.
2. False self-presentation on Instagram would be indirectly associated with consideration of cosmetic surgery through BICP. That is, individuals who engage in more body image control behaviors online would be more likely to consider engaging in cosmetic enhancements of their appearance to control their body image offline.
3. False self-presentation on Instagram would be indirectly related to the consideration of cosmetic surgery through photo manipulation and body shame operating in serial. That is, individuals who enhance their photos to impress others would be less satisfied with their appearance, thus experiencing body shame. These individuals would then be more likely to search for potential remedies to alleviate their discomfort, such as cosmetic surgery.

Furthermore, considering the broad demand for a more comprehensive approach to the relationship between social media use and body image regarding gender [43, 44] and the fact that an increasing number of men are undergoing cosmetic procedures [45], we believe that it is necessary to include a male sample in this research.

## Methods

### Participants

Our online survey was accessed by 748 participants; however, only 497 individuals were included in the final sample. Of the 251 participants removed from the analysis, 216 (28.8%) did not complete the questionnaire, 30 (4%) were older than 30, and 5 (0.7%) completed the survey in less than three standard deviations below the average time taken by all the respondents. Outliers were checked using the Mahalanobis distance [46], and no significant observations were found. The final sample consisted of 497 Italian-speaking individuals (28.2% males), aged 18–30 (M = 22.5, SD = 2.73). Most of the participants finished secondary school (*n *= 232, 46.7%) or earned a bachelor’s degree (*n* = 195, 39.2%). Regarding occupational status, the majority of participants reported being students (*n *= 365, 73.4%).

### Measures

#### False Self-presentation on Instagram

The compare/impress subscale of the self-presentation on Facebook Questionnaire [47] was used to assess the extent to which people manipulated their self-presentation on Instagram to impress others. This scale was translated from English to Italian using a standard translation/back-translation process. Consistent with Jackson and Luchner [48], we adapted this measure by replacing the word “Facebook” with “Instagram.” This subscale consists of three items (e.g., “I only show the aspects of myself on Instagram that I know people would like”), for which participants responded on a scale ranging from 1 (strongly disagree) to 5 (strongly agree). Scores were averaged, with higher scores indicating greater feelings of false self-presentation on Instagram. Cronbach’s alpha was .68 (95% CI .62, .73).

#### BICP

The Italian version of the Body Image Control in Photos Questionnaire—revised [21] was used to evaluate participants’ photo management and control online and offline. This scale consists of 16 items, covering different behaviors: selfie-related behaviors (e.g., “I take photos of myself several times, until I obtain the image that makes me appear more attractive, in order to post it on my profile”), privacy filter behaviors (e.g., “I use privacy filters because I don’t want some people to see photos in which I have not come out very well”), positive body image (e.g., “I post those photos which I hope will receive praise for my appearance”), sexual attraction behaviors (e.g., “I take provocative photos in order to attract others’ attention to me”), and negative body image (e.g., “I feel awkward if I notice that someone has posted photos that show my body’s defects”). Participants responded to each item on a scale ranging from 1 (never) to 5 (always). Scores were averaged, with higher scores indicating greater photo control. Cronbach’s alpha was .88 (95% CI .87, .89).

#### Photo Manipulation

The Italian version of the photo manipulation scale [27] was used to evaluate how often participants manipulated and edited photos of themselves before sharing them on SNSs. This scale consists of eight items covering three dimensions: photo filter use (e.g., “How often do you use interactive filters such as the dog’s snout and flower crown?”), body image manipulation (e.g., “How often do you make yourself look skinnier?”), and facial image manipulation (e.g., “How often do you edit or use apps to smooth skin?”). Participants responded to each item on a scale ranging from 1 (never) to 5 (always). Scores were averaged, with higher scores indicating greater use of photo manipulation techniques. Cronbach’s alpha was .75 (95% CI .71, .79).

#### Body Shame

The shame subscale of the Italian version of the Objectified Body Consciousness Scale [19] was used to assess the degree to which individuals feel shame about their bodies and appearance. This subscale consists of eight items (e.g., “I feel ashamed of myself when I haven’t made the effort to look my best”), to which the participants responded on a scale ranging from 1 (strongly disagree) to 7 (strongly agree). Scores were averaged, with higher scores indicating higher levels of self-reported body shame. Cronbach’s alpha was .87 (95% CI .85, .88).

#### Consideration of Cosmetic Surgery

The consider subscale of the Italian version of the Acceptance of Cosmetic Surgery Scale [49] was used to measure participants’ cosmetic surgery intentions. This subscale consists of five items (e.g., “In the future, I could end up having some kind of cosmetic surgery”), to which the participants responded on a scale ranging from 1 (strongly disagree) to 7 (strongly agree). Scores were averaged, with higher scores indicating higher consideration for cosmetic surgery. Cronbach’s alpha was .92 (95% CI: .91, .93).

#### Time Spent on Instagram

Time spent on Instagram was assessed with the question “How much time in a day do you spend on Instagram?” with answers ranging from less than 15 min to more than 4 h. Answers were then adapted on a decimal scale from 0 to 4 where a difference of 0.25 points represented a 15-minute time span.

#### Body Surveillance

The surveillance subscale of the Italian version of the Objectified Body Consciousness Scale [19] was used to measure participants’ behavioral tendencies toward body surveillance or preoccupation with monitoring their appearance, which served as a control variable in our analyses. This subscale consists of eight items (e.g., “During the day, I think about how I look many times”), to which the participants responded on a scale ranging from 1 (strongly disagree) to 7 (strongly agree). Scores were averaged, with higher scores indicating higher levels of self-reported body surveillance. Cronbach’s alpha was .80 (95% CI .77, .83).

### Procedures

The study was conducted through an online survey administered via the Qualtrics platform (secure online data collection software), available from March to April 2022. Participants were eligible if they were between 18 and 30 years old and possessed an Instagram account. Respondents were recruited using opportunistic sampling techniques, such as social media (WhatsApp, Instagram, Facebook) and snowball sampling. Participation in the study was voluntary. Before completing the survey, participants were provided with general information about the research purpose and the scales administered. The anonymous nature of the data collected was reported within the informed consent, as well as the possibility of withdrawing from the study at any time. The study was approved by the Ethical Committee for Psychological Research at the University of Padova. All procedures performed in studies involving human participants were in accordance with the ethical standards of the institutional and/or national research committee and with the 1964 Helsinki Declaration and its later amendments or comparable ethical standards.

### Data Analysis

Statistical analyses were performed using the statistical program R [50] and the *lavaan* package [51]. First, construct validity of all scales was controlled using confirmatory factor analysis, with all measures showing acceptable internal consistency. Second, descriptive statistics and intercorrelations between all variables were calculated. Second, path analysis was used to test the hypothesized model^1^ (Fig. 1). Independent variables were allowed to co-vary as dependent variables. A bootstrapping procedure with 5000 samples was used to test the presence and size of the expected mediation [52]. To evaluate the overall model accuracy of fit, several indices were used: the comparative fit index (CFI), whose value can range from 0 to 1 fit and where values higher than 0.95 are suggested for a good fit [53]; the standardized root mean square residuals (SRMR), for which values lower than 0.08 are recommended [53]; and the total coefficient of determination (TCD), which measures the overall proportion of variance explained by the model [54]. In the tested model, false self-presentation was expected to be positively associated with photo manipulation both directly and indirectly through the mediation of BICP. Simultaneously, BICP mediates the relationship between false self-presentation and consideration of cosmetic surgery. Moreover, photo manipulation is positively associated with the consideration of cosmetic surgery both directly and indirectly through the mediation of body shame. Gender, time spent on Instagram, and body surveillance were used as control variables for all the constructs.

## Results

Descriptive statistics were used to assess the means, standard deviations, skewness, kurtosis (Table 1), and intercorrelations between the variables (Table 2). Regarding data normality, all the variables were within the range suggested by West et al. [55] for kurtosis (absolute value  < 7) and skewness (absolute value  < 2).

**Table 1:** Mean (M), standard deviation (SD), range skewness, kurtosis, and intercorrelation between the variables

Variable	1	2	3	4	5	6	M (SD)
1. Time on Instagram							1.42 (.89)
2. Body surveillance	.17***						4.37 (1.06)
3. False self-presentation	.12**	.56***					2.84 (.95)
4. BICP	.23***	.60***	.60***				2.37 (.72)
5. Photo manipulation	.29***	.39***	.41***	.57***			1.77 (.58)
6. Body shame	.12*	.59***	.41***	.51***	.35***		3.79 (1.39)
7. Consideration of cosmetic surgery	.18***	.34***	.23***	.35***	.29***	.28***	3.11 (1.79)

*N*= 504; BICP, body image control in photos

^***^p < .001

^**^p < .01

^*^p < .05

**Table 2:** Standardized indirect effects of the independent variable (false self-presentation) on the outcome (consideration of cosmetic surgery) via the mediators (BICP, photo manipulation, and body shame)

Independent variable	Mediators	Outcome			
		Consider			
		Beta	SE	*z*	*p*
False self-presentation	BICP	.049	.044	2.086	.037
	BICP → photo manipulation	.014	.018	1.419	.156
	BICP → photo manipulation → body shame	.001	.003	.903	.366
	Photo manipulation	.009	.014	1.220	.223
	Photo manipulation → body shame	.001	.002	.767	.443

N= 504; BICP, body image control in photos; Consider, consideration of cosmetic surgery

Table 1 shows that all variables were positively associated. False self-presentation was associated with BICP and photo manipulation, with the latter being positively correlated with body shame and consideration of cosmetic surgery. Body shame was associated with BICP showing a large effect size. Body surveillance was associated with all the considered variables, with effect sizes ranging from medium to large, supporting the decision to implement it as a control variable in the model. Lastly, gender and time spent on Instagram were correlated with all the variables, but their effect sizes were small.

**Fig. 2:**
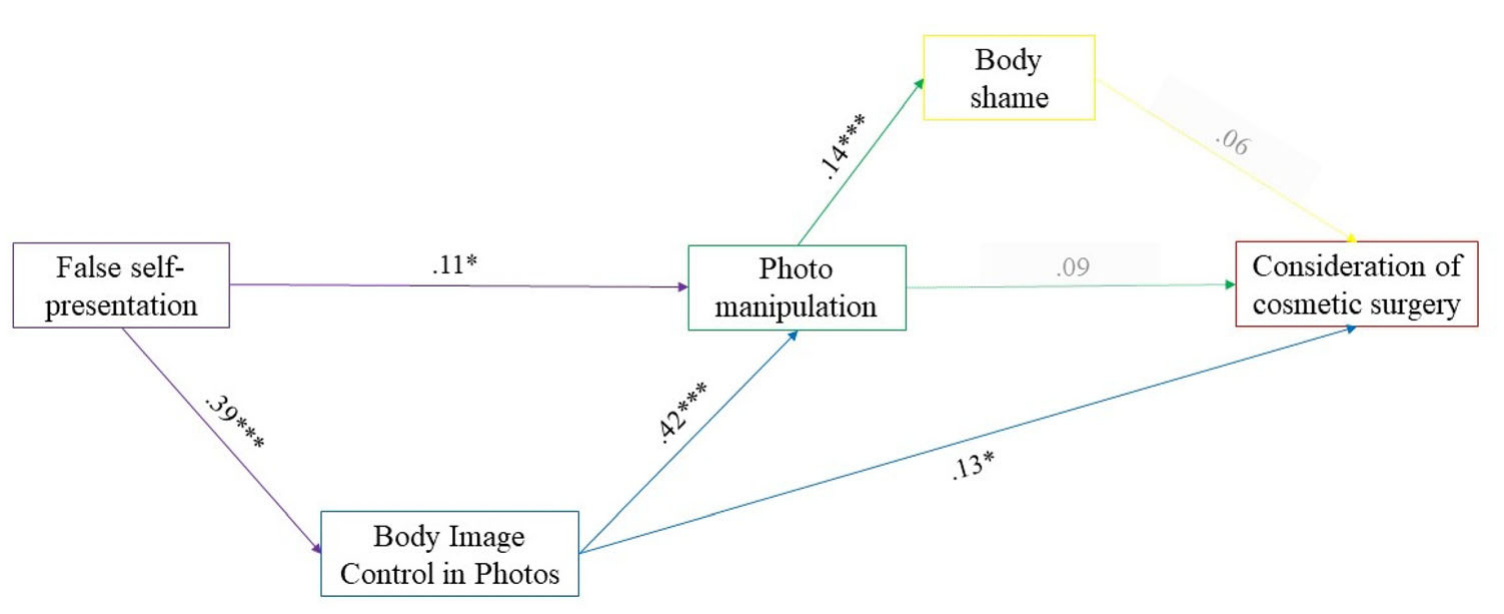
Standardized parameters for the model. *Note:* For clarity, control variables, covariances, residual variances, and mediation effects are not shown. *N* = 497. *p < .05; **p < .01; ***p <.001

The path coefficients are shown in Fig. 2. As expected, false self-presentation was positively associated with both BICP (*β *= .39, *p* < .001) and photo manipulation (*β *= .11, *p *= .020), while BICP was positively associated with both photo manipulation (*β *= .42, *p* < .001) and consideration of cosmetic surgery (*β *= .13, *p* = .038). Photo manipulation was associated with body shame (*β* = .14, *p* = .001), but not with consideration of cosmetic surgery (*β* = .09, *p* = .131). Finally, body shame did not show a significant association with consideration of cosmetic surgery (*β* = .06, *p* = .306). Regarding the control variables, gender and time spent on Instagram showed significant associations with BICP (*β* = .17, *p* < .001) (*β* = .09, *p* = .007) and photo manipulation (*β* = .11, *p* = .006) (*β* = .15, *p* = .001), but not with false self-presentation (*β* = − .02, *p* = .664) (*β* = .03, *p* = .446), body shame (*β* = .04, *p* = .320) (*β* = − .02, *p* = .603), and consideration of cosmetic surgery (*β* = .09, *p* = .051) (*β* = .08, *p* = .093). On the other hand, body surveillance was positively associated with false self-presentation (*β* = .56, *p* < .001), BICP (*β* = .33, *p* < .001), body shame (*β* = .53, *p* < .001), and consideration of cosmetic surgery (*β* = .17, *p* = .002), but not with photo manipulation (*β* = .03, *p* = .493). All the indirect effects are presented in Table 2.

Regarding the three hypothesized mediation paths, the first one was significant, with false self-presentation being positively associated with photo manipulation through BICP (*β* = .16, *p* < .001). Furthermore, BICP mediated the relationship between false self-presentation and consideration of cosmetic surgery (*β* = .05, *p* = .039). Contrary to the initial hypothesis, photo manipulation and body shame did not mediate the relationship between false self-presentation and cosmetic surgery intentions (*β* = .001, *p* = .443).

The statistical model fit the data well [CFI = .99; SRMR = .02]. The TCD was .62 (corresponding to a correlation of *r* = .79), which is a large effect size according to the traditional criteria of Cohen [56]. Furthermore, the statistical model accounted for most of the variance in BICP (51%) and a reasonable percentage of the variance in false self-presentation (32%), photo manipulation (37%), body shame (37%), and acceptance of cosmetic surgery (18%).

## Discussion

In the current study, we provide useful insights into the relationship between false self-presentation on Instagram and consideration of cosmetic surgery, as well as its underlying mechanisms. Our findings show that when young adults present themselves in a disingenuous way to impress others, they might become more involved in photo manipulation, which in turn would increase the amount of shame they feel about their bodies. Contrary to common beliefs though, neither body shame nor photo manipulation is the reason why people consider cosmetic procedures; that is, users seem more preoccupied with keeping up their online façade in the offline world as well.

Our sample stated that they spend a little less than 1 hour and a half on Instagram. Participants reported higher levels of false self-presentation, body surveillance, and body shame in comparison to previous studies [47] and to the Italian version of the Objectified Body Consciousness Scale [19], but they showed lower levels of photo manipulation behaviors [27]. Descriptive statistics regarding BICP and acceptance of cosmetic surgery are similar to those of previous scale validations [21, 49].

Consistent with our first hypothesis, false self-presentation on Instagram is directly and indirectly associated with photo manipulation through the mediating effect of BICP. Individuals who feel the need to be popular might engage in photo manipulation, because it is an accessible way to improve their self-presentation [23]. In addition, these behaviors can be implemented both by editing individual photos and by exerting a much more generalized control of body image across social media. To this regard, people who use their photos in an instrumental way may exercise greater control over their body image and show greater investment toward visual activities to put forth a better portrait of themselves [27]. It is also possible that Instagram users feel obliged to post manipulated photos to impress others due to the constant pressure to present only the best part of themselves on the platform [3]. Nevertheless, Instagram’s affordances incentivize individuals to focus on their body image presentations because photos that conform more to beauty norms are better received [17].

Consistent with our second hypothesis, false self-presentation on Instagram is indirectly linked to consideration of cosmetic surgery through the mediating effect of BICP. That is, people who engage in more controlling behaviors related to their body image self-presentation online are more likely to consider ways to control their body image offline. A plausible explanation for this phenomenon is rooted in the self-effect theory [57], which states that individuals infer their self-concept by retrospectively observing their behaviors. By noticing that their virtual persona approves and uses body image control practices, individuals may subconsciously infer that their offline self will endorse similar habits. Therefore, they will show a greater consideration of cosmetic procedures, as they are portrayed as an easy and accessible way to control how they present themselves to others [40].

Another plausible explanation for this association resides in how cosmetic procedures are perceived today. The medicalization of appearance, the normalization of cosmetic surgery, and its popularity have reversed the significance of cosmetic enhancements. While they were once seen as a way to shield oneself from undesirable future selves [42], they are now an accessible way to manage one’s self-presentation. Thus, people who feel the need to present themselves as conforming to beauty ideals might see cosmetic surgery as a possible and accessible solution to reach them.

Our third hypothesis was only partially supported, with photo manipulation being associated with body shame, although the latter is not related to the consideration of cosmetic surgery. This relationship might be explained by the fact that people who edit their photos need to take an external point of view to understand what needs editing and effectively engage in self-objectification [58], thus eliciting higher feelings of body shame.

The fact that neither photo manipulation, nor body shame was associated with cosmetic surgery intentions is surprising as it contradicts previous research [34, 35, 59]. This discrepancy might be caused by different reasons. Research shows that not all kinds of photo manipulation are related to body dissatisfaction: “normative” behaviors such as little blemish removal or light changes do not seem to have great effects compared to facial filters and body manipulation [44, 60]. To this regard, the Photo Manipulation Scale used in this study assessed overall strategies of photo editing, while Maes and de Lenne [35], for instance, showed that only the use of face filters was associated with increased consideration of cosmetic surgery. Furthermore, in line with uses and gratification theory [61] individual’s motivations represent a fundamental aspect regarding potential outcomes of social media use. It’s possible that some people may be engaging with Instagram for the specific purpose of showing-off, while others may be using the platform for other reasons. Both groups of individuals may engage in photo manipulation, but only the former group is likely at a greater risk for incurring in potential risks [62]. In this regard, it is plausible to think that reports calling for the hazardous nature of Instagram’s filter fail to consider the role of user’s motivation.

The current study has some limitations. First, its cross-sectional nature precludes any conclusions regarding the causal nature of the relationships between constructs. Future studies (e.g., experimental, or longitudinal studies) are needed to prove the direction of any causal relationships hypothesized in this study. Second, our research relied on self-report measures. Even though this approach is widely used, its validity is often scarce as users tend to underestimate their social media use [63]. Third, all the respondents were young Italian adults; thus, the findings may not be representative of other demographics who might engage in these behaviors (e.g., adolescents). Furthermore, the sample presented a sex imbalance, with women comprising over 70% of the total participants. Future studies should explore how these constructs are related to different populations using more homogeneous samples.

## Conclusion

Our results confirm that users’ motivations are important predictors of their online behaviors. This study provides a new perspective through which cosmetic surgeons can examine patients’ motivations. In particular, surgeons should try to understand if individuals are considering cosmetic procedures just to keep up with their online façade in the offline world or if they show a serious need to embark on these treatments. Otherwise, if people are willing to undertake cosmetic procedures to impress others in real life, they may repeat these procedures if they feel that the results are no longer satisfying, thus risking major complications [13]. This may already be true, with anecdotical evidence showing that repeatable and accessible non-invasive procedures, such as Botox and injectables, are in high demand [64]. From a practical point of view, surgeons should fully examine patients’ motivations and expectations before providing them with services. In fact, if individuals modify their bodies without changing attitude toward their body image, concerns about their appearance may not decrease, leaving them discomfortable with their own body image [65].

Regarding Instagram use, engaging with the platform to boost one’s reputation by impressing others might be a risk factor for developing body shame. People should try to present themselves in a less controlled way online to reduce this. In this context, intervention programs might encourage users to present a more authentic version of themselves online to steer clear of self-objectification and improve their social media skills [66].

## Footnotes

1 An exploratory multigroup analysis was performed to test the equivalence of the model across male and female subsamples, and it is available upon request.
